# Prevalence of Esophageal Dysmotility and Reflux in Muscle Tension Dysphonia Patients

**DOI:** 10.1002/lary.70274

**Published:** 2025-11-26

**Authors:** Megan Brianne Saltsgaver, Madison Cheung, Theo Covello, Weihai Zhan, Inna A. Husain

**Affiliations:** ^1^ University of Illinois College of Medicine Rockford Rockford Illinois USA; ^2^ Indiana University School of Medicine South Bend South Bend Indiana USA; ^3^ Powers Health Munster Indiana USA

**Keywords:** barium esophagram, esophageal dysmotility, laryngopharyngeal reflux, muscle tension dysphonia

## Abstract

**Objectives:**

Laryngopharyngeal reflux (LPR) contributes to muscle tension dysphonia (MTD); however, symptoms overlap with esophageal dysmotility. Study aims were to determine the prevalence of esophageal disorders among MTD patients and correlate pathologies to patient‐reported outcome measures (PROMs).

**Methods:**

Retrospective chart review of MTD patients diagnosed by laryngologists who underwent barium esophagram for suspected LPR. Demographics, Voice Handicap Index 10 (VHI‐10), Voice Related Quality of Life (V‐RQOL), and imaging were reviewed. The Kruskal–Wallis test was used to compare VHI‐10 and V‐RQOL across groups. The Wilcoxon rank‐sum test was applied to compare scores including their subitems, between dysmotility only and reflux only groups.

**Results:**

A total of 128 patients with MTD underwent barium esophagram. The average age was 61 years; 70% were female. Esophageal findings included dysmotility only (31%), reflux only (24%), both (12%), and neither (33%). The reflux‐only group reported significantly worse voice‐related outcomes on PROMS compared to dysmotility only. Reflux‐only patients had higher total VHI‐10 (*p* = 0.04) and were more likely to report difficulty being heard (*p* = 0.01), understood in noisy environments (*p* = 0.02), restricted socially (*p* < 0.01), and left out of conversations (*p* = 0.03). V‐RQOL items like speaking loudly (*p* = 0.04), using phone (*p* = 0.04), performing work duties (*p* = 0.03), and feeling less outgoing (*p* = 0.02) were also more negatively impacted in reflux‐only.

**Conclusion:**

Esophageal dysmotility is prevalent in MTD patients and may mimic LPR. Patients with reflux may have more negative impacts on V‐RQOL. Although esophagrams can be used as a screening tool, further studies can help elucidate the connection between esophageal dysmotility and laryngeal function.

**Level of Evidence:**

3.

## 1 Introduction

Muscle tension dysphonia (MTD) is a collective term for disorders of increased laryngeal musculature tension leading to abnormal vocal fold behavior [1]. Symptoms include vocal fatigue, voice loss, hoarseness, vocal strain, pain, vocal tract irritability or dryness, neck tightness, or globus sensation [1]. Laryngopharyngeal reflux (LPR) is often thought to be a contributor to MTD [2]. Laryngopharyngeal reflux disease (LPRD) is a disease of the upper aerodigestive tract resulting from the direct and indirect effects of gastric contents on the larynx, pharynx, and nasal cavity inducing morphological or neurological changes [3]. In the direct mechanism, regurgitated gastroduodenal contents including hydrochloric acid, bile, pepsin, and trypsin are proposed to cause changes to the pharynx and be the cause of symptoms patients with LPR experience [4]. Regurgitation via LPR is proposed to lead to secondary MTD by the body's airway protection mechanisms, including closure of the glottis, coughing or choking, and tightening of laryngopharyngeal constrictor muscles [1]. Proton pump inhibitors (PPIs) are commonly given as treatment in patients with MTD due to presumed underlying LPR, although a study showed 70.3% of patients continued to experience hoarseness and associated throat complaints on PPI treatment, suggesting acid suppression may not control LPR, inefficient voice patterns persist despite LPR management, or other conditions may exist that affect patient response to treatment [5].

One such condition in which clinical presentation often overlaps with that of LPR is esophageal dysmotility. Esophageal dysmotility has been established to be prevalent among patients with LPR symptoms, including esophagogastric junction outflow or major peristaltic disorder [6]. Knight et al. observed that 73% of patients with LPR symptoms had abnormal esophageal motility on esophageal manometry [7]. Symptoms may arise via the indirect mechanism of LPRD, via vagal nerve activation, or contribute to reflux due to poor esophageal clearance [4].

The objective of this study was to determine the prevalence of reflux and esophageal dysmotility disorders among MTD patients on screening esophagram and identify any correlation between the presence of dysmotility or reflux and voice‐related patient‐reported outcome measures (PROMs). We hypothesize that a subset of patients with MTD presumed to be caused by LPR may actually have underlying esophageal dysmotility, contributing to their symptoms. Identifying esophageal motility disorders in these patients may allow practitioners to offer more targeted treatment plans.

## 2 Methods

### 2.1 Participants

This was a retrospective chart review of all adult patients with MTD who were clinically suspected to have LPRD by a laryngologist who underwent double contrast barium esophagram as part of their workup from February 2023 to June 2024. Patients with a history of neurological disorders or prior gastrointestinal surgery were excluded from this study. Demographics, PROMs, radiology results, and esophagogastroduodenoscopy reports were reviewed. This study was approved through Powers Health Institutional Review Board.

PROMs analyzed included the VHI‐10, and V‐RQOL collected during the initial laryngology visit. Total scores and individual questions from each survey were evaluated.

### 2.2 Statistical Analysis

Data distribution was assessed using visual and statistical methods, including histograms, skewness, kurtosis, and the Shapiro–Wilk test, to determine whether the data were normally or approximately normally distributed. These assessments indicated that the data did not meet the assumptions of normality. As a result, nonparametric statistical methods were employed for group comparisons. Specifically, the Wilcoxon rank‐sum test was used to evaluate differences in scores between two independent groups, while the Kruskal‐Wallis test was applied to compare scores across four distinct groups: (1) no dysmotility and no reflux, (2) reflux only, (3) dysmotility only, and (4) both dysmotility and reflux. These nonparametric tests are appropriate for ordinal or non‐normally distributed continuous data and do not require the assumption of homogeneity of variances. All statistical tests were two‐tailed, and a significance level (*α*) of 0.05 was used to determine statistical significance. Statistical analyses were conducted using SAS software, version 9.4 (SAS Institute Inc., Cary, NC).

## 3 Results

There were 128 patients evaluated by a laryngologist with clinically suspected secondary MTD due to LPRD that underwent barium esophagram. Out of the 128, there were 89 patients identifying as female, with a mean age of 60.9, and white/Caucasian patients making up 73.4% of the population. Forty‐three percent (*N* = 55) had the presence of dysmotility on their esophagram.

There were 40 (31%) esophagrams showing dysmotility only, 31 (24%) with reflux only, 15 (12%) with dysmotility and reflux, and 42 (33%) with no dysmotility or reflux. There was no significant difference in total raw V‐RQOL or VHI‐10 scores among dysphonia patients that had dysmotility only, reflux only, dysmotility and reflux, or findings of neither (Table 1).

**TABLE 1:** Comparisons among 128 patients with muscle tension dysphonia.

Groups	VHI‐10	V‐RQOL
No dysmotility, no reflux (*n* = 42)	12.2 ± 10.7, 10.5	21.2 ± 9.6, 19.0 (72)
Reflux only (*n* = 31)	14.2 ± 10.6, 12.0	21.5 ± 9.7, 20.0 (71.25)
Dysmotility only (*n* = 40)	8.3 ± 9, 4.0	16.9 ± 9.7, 13.0 (82.75)
Both dysmotility and reflux (*n* = 15)	12.6 ± 12.6, 9.5	18.8 ± 11.6, 13.0 (78)
*p*	0.18	0.14

*Note*: Mean ± SD, median. *p* values were obtained using the Kruskal–Wallis tests. V‐RQOL raw scoring; in parentheses standard scoring.

When comparing patients with dysmotility only (*n* = 40) to patients with reflux only (*n* = 31), the reflux group reported significantly greater vocal handicap. They had higher total VHI‐10 scores (14.2 ± 10.6 vs. 8.3 ± 9.0; *p* = 0.04) and were more likely to report difficulties being heard (*p* = 0.01), being understood in noisy environments (*p* = 0.02), voice‐related restrictions in personal/social life (*p* < 0.01), and feeling left out of conversations (*p* = 0.03, Table 2).

**TABLE 2:** Comparisons of VHI‐10 score and subitems between dysmotility only and reflux only (*n* = 71).

	Dysmotility only	Reflux only	*p*
VHI‐10 Total	8.3 ± 9.0, 4.0	14.2 ± 10.6, 12.0	0.04
Voice makes it difficult to be heard	0.9 ± 1.2, 0.0	2.0 ± 1.5, 2.0	0.01
Difficulty being understood in noisy room	0.9 ± 1.2, 0.0	1.8 ± 1.3, 2.0	0.02
Voice restricts personal and social life	0.5 ± 1.0, 0.0	1.7 ± 1.5, 2.0	< 0.01
Feeling left out of conversations	0.5 ± 1.1, 0.0	1.0 ± 1.3, 1.0	0.03
Voice causes issues with income	0.1 ± 0.2, 0.0	0.2 ± 0.7, 0.0	0.67
Strain to produce voice	1.1 ± 1.4, 0.0	1.4 ± 1.4, 1.0	0.44
Voice clarity is unpredictable	1.5 ± 1.4, 1.0	2.0 ± 1.5, 2.0	0.24
Voice upsets me	1.1 ± 1.5, 0.0	1.9 ± 1.5, 2.0	0.07
Voice makes me feel handicapped	0.6 ± 1.2, 0.0	1.0 ± 1.3, 0.0	0.11
People ask “What's wrong with your voice?”	1.2 ± 1.5, 0.0	1.4 ± 1.4, 1.0	0.48

*Note*: Mean ± STD, median. *p* values were obtained using Wilcoxon rank‐sum test.

On the V‐RQOL, the reflux group reported a lower V‐RQOL. Although total V‐RQOL scores were higher in the reflux group (21.5 ± 9.7 vs. 16.9 ± 9.7), the difference did not reach statistical significance (*p* = 0.07). However, the reflux group was significantly more likely to report trouble speaking loudly or being heard (*p* = 0.04), not knowing about what will come out when speaking (*p* = 0.04), trouble using the phone (*p* = 0.04), trouble doing jobs or practicing professions because of voice (*p* = 0.03), and being less outgoing due to voice (*p* = 0.02, Table 3).

**TABLE 3:** Comparisons of V‐RQOL score and subitems between dysmotility only and reflux only (*n* = 71).

	Dysmotility only	Reflux only	*p*
V‐RQOL total	16.9 ± 9.7, 13.0	21.5 ± 9.7, 20.0	0.07
Trouble speaking loudly or being heard	2.0 ± 1.3, 2.0	2.7 ± 1.1, 3.0	0.04
Run out of air when talking	2.0 ± 1.2, 2.0	2.6 ± 1.6, 3.0	0.29
Not knowing what will come out when speaking	1.7 ± 1.2, 1.0	2.4 ± 1.3, 2.0	0.04
Anxious or frustrated by voice	2.0 ± 1.4, 1.0	2.2 ± 1.4, 2.0	0.55
Depressed because of voice	1.6 ± 1.3, 1.0	2.0 ± 1.4, 1.0	0.16
Trouble using phone	1.4 ± 0.9, 1.0	2.1 ± 1.3, 2.0	0.04
Trouble with job	1.2 ± 0.7, 1.0	1.6 ± 0.8, 1.0	0.03
Avoid going out	1.3 ± 0.9, 1.0	1.7 ± 1.1, 1.0	0.14
Have to repeat myself	1.9 ± 1.3, 1.0	2.3 ± 1.3, 2.0	0.21
Less outgoing due to voice	1.3 ± 1.0, 1.0	2.0 ± 1.2, 1.0	0.02

*Note*: Mean ± STD, median. *p* values were obtained using Wilcoxon rank‐sum test.

## 4 Discussion

MTD is a functional voice disorder with multifactorial etiology, including psychological stress, vocal overuse, age‐related changes, and LPR [1, 8, 9, 10, 11]. While voice therapy remains a mainstay of treatment for MTD and has demonstrated favorable outcomes, optimal management requires accurate identification of underlying etiologies [5, 12, 13, 14, 15]. Our study aimed to characterize the prevalence of esophageal dysmotility and reflux among patients with MTD secondary to suspected LPRD. We found that 43% demonstrated esophageal dysmotility on barium esophagram, with 31% of those cases occurring without evidence of reflux. This suggests that esophageal dysmotility may be prevalent and an underrecognized contributor to MTD symptomatology.

Recent studies suggest that esophageal dysmotility may be an underrecognized contributor to voice symptoms often attributed to LPR. Up to 73% of patients with LPR‐like symptoms demonstrate abnormal esophageal motility on manometry [7]. Our findings align with this, revealing a 43% prevalence of dysmotility in MTD patients assessed by barium esophagram. Given that esophageal dysfunction can provoke laryngeal protective mechanisms–such as glottic closure, increased tension and chronic coughing–esophageal dysmotility may mimic or exacerbate MTD even in the absence of true reflux [1].

Previous studies have analyzed the Reflux Symptom Index (RSI) with MTD patients reporting higher RSI scores in the absence of objective LPR findings, suggesting a disconnect between symptom perception and physiological evidence of reflux [16]. This mismatch may reflect alternative mechanisms such as esophageal dysmotility or heightened laryngeal sensitivity. Additional studies have emphasized that hoarseness and other laryngeal complaints attributed to LPR may be from alternative etiologies only identifiable through comprehensive laryngeal examination, including videostroboscopy [17]. These studies help highlight the need for comprehensive evaluation strategies in MTD patients, including visualization of the larynx and esophageal testing, particularly when subjective reflux symptoms are not matched by objective findings or prolonged.

This study demonstrates the use of the barium esophagram, an easy, readily available test that can provide diagnostic information for suspected LPR patients. Although there have been previous studies supporting the prevalence of esophageal dysmotility in patients with LPR symptomatology, the role of barium esophagrams in evaluating MTD patients remains underreported.

Nearly half of patients with MTD and suspected LPRD demonstrated both esophageal dysmotility and reflux on barium esophagram. This finding is clinically meaningful, as persistent MTD symptoms despite reflux management and voice therapy may be influenced by underlying esophageal dysfunction. Notably, 31% of patients exhibited esophageal dysmotility in the absence of reflux. While barium esophagrams are not the gold standard for diagnosing reflux, these findings further underscore the potential role of esophageal function in the clinical presentation of MTD.

A secondary aim was understanding the relationship between the presence of esophageal dysmotility and other voice‐related outcome measures, specifically VHI‐10 and V‐RQOL. There were no significant differences in total VHI‐10 or V‐RQOL scores between patients with esophageal dysmotility only and other groups (i.e., reflux only, dysmotility + reflux, or no reflux and no dysmotility). Although the difference in total V‐RQOL scores between patients with dysmotility alone and those with reflux alone did not reach statistical significance (*p* = 0.07), several subitems, such as trouble speaking loudly, uncertainty about what will come out when speaking, trouble using the phone, trouble with job performance, and being less outgoing, were significantly worse in the reflux group, suggesting that patients with dysmotility alone may perceive somewhat better V‐RQOL overall. Whereas esophageal dysmotility may contribute to symptoms via vagally mediated reflexes, reflux likely contributes to dysphonia through direct laryngeal tissue injury. It is possible that direct tissue injury results in a more severe impact on V‐RQOL [4].

Diagnosis of LPR is notoriously difficult due to the lack of ability to detect reflux in real‐time. The HEMII‐pH is the gold standard for identifying LPR due to its ability to note gaseous reflux, characterize acidity of the refluxate, and correlate symptoms with events. However, the use of hypopharyngeal probes is not routine, often not offered by otolaryngology offices diagnosing patients with LPR, and can be invasive and uncomfortable [3].

High‐resolution manometry (HRM), although considered the gold standard for diagnosing esophageal motility disorders, has its drawbacks. The placement of probes down the esophagus creates suboptimal testing conditions and the sophisticated software required for interpretation makes it the pricier option, leaving it less accessible [8]. Barium esophagram, while considered inferior to HRM for evaluating esophageal dysmotility, offers some practical advantages. It can detect real‐time bolus reflux, esophageal dysmotility, and bolus stasis—all of which may contribute to LPRD symptoms. Downsides include the use of contrast, fluoroscopy radiation, and the lack of ability to correlate symptoms with the dysmotility or reflux seen [18, 19].

Despite barium esophagrams' limitations, they are more accessible, more affordable and better tolerated than HRM. Our study challenges the statement that barium esophagrams are poor screening tests. With 86 abnormal esophagrams, this modality can detect not just motility disorders, but also stasis, reflux, and anatomical anomalies which may lead to LPRD symptoms. This makes barium esophagram an acceptable addition in the first steps in the workup of patients with suspected LPR to determine other possible etiologies [18, 19].

This study does have a few limitations, the most notable being its retrospective design and the risk of selection bias. Although all patients were offered a barium esophagram for a suspicion of LPR, this analysis only includes those who completed the study—possibly including patients who had more severe symptoms and were willing to undergo further testing. HRM was not used to confirm dysmotility in patients with dysmotility identified on barium esophagram. Additionally, this study included individuals who were presumed to have secondary MTD based on clinical suspicion of reflux as a trigger. As part of their diagnostic workup, patients underwent barium esophagram to evaluate for potential reflux or esophageal dysmotility as contributing factors. It is also possible that other contributors to MTD, such as allergic rhinitis, were present but not fully accounted for. This may limit the generalizability of our findings and highlights the need for more detailed phenotyping in future prospective studies. Additionally, our study did not control for patients actively on LPRD or GERD management such as acid suppression or alginate therapy nor could it offer definitive correlation of the esophageal findings and patient‐reported laryngopharyngeal symptoms. Lastly, this study did not include formal adjustments for multiple comparisons (e.g., Bonferroni correction). This was an intentional decision, given the exploratory nature of the analysis and the limited sample size. In this context, strict correction methods could substantially reduce statistical power, increasing the risk of Type II errors and potentially obscuring clinically meaningful findings. Our primary goal was to identify potential signals for future investigation rather than to make definitive inferential claims. Future confirmatory studies with larger sample sizes and appropriate statistical corrections will be necessary to validate these preliminary findings.

Despite these limitations, our evidence suggests that esophageal dysmotility is prevalent among MTD patients suspected to have LPR. At 43%, this compares to several other studies using HRM to identify dysmotility when LPR was suspected, making barium esophagrams an acceptable, cheaper, and more accessible first option in the workup of patients suspected to have LPRD [20].

A study correlating HRM or hypopharyngeal‐esophageal multichannel intraluminal impedance‐pH monitoring (HEMII‐pH) results to patient‐reported outcomes may better elucidate a screening tool for LPR [21, 22]. Although there is good correlation with PROMs such as the reflux symptom score, PROMs are not considered diagnostic tools and previous studies have demonstrated higher patient satisfaction when a diagnosis of LPR can be obtained [23]. While not a definitive test, the barium esophagram offers valuable data such as identifying reflux, dysmotility, and structural anomalies such as hiatal hernias, cricopharyngeal bars, and strictures that can guide treatment. In this way, it serves as a useful and underutilized tool in the broader evaluation of patients with complex laryngopharyngeal symptoms.

## 5 Conclusion

LPR is a common diagnosis for secondary MTD and esophageal dysmotility has been shown to be prevalent in patients with LPR symptomatology [7, 24]. In our study, 43% of patients suspected to have LPR symptoms also demonstrated esophageal dysmotility on esophagram. Given the high prevalence of esophageal dysmotility in our cohort, these findings support the indirect mechanism of LPRD, where esophageal motility disorders may mimic or contribute to LPR‐like symptoms.

Interestingly, patients with esophageal dysmotility in our study tended to report fewer voice‐related symptoms, which may suggest that this subgroup of LPRD, indirect LPRD, represents a less severe or distinct phenotype of MTD. This highlights the potential heterogeneity within MTD populations and underscores the importance of tailored diagnostic evaluation.

Since nearly half of participants had dysmotility, and many lacked traditional reflux findings, our results support consideration of diagnostic evaluation for LPRD before initiating empiric treatment. Future studies should investigate LPRD subsets in MTD patients using advanced diagnostic tools such as HRM and HEMII‐pH, and evaluate whether symptom‐targeted treatments based on esophagram findings can improve voice outcomes.

## Funding

The authors have nothing to report.

## Disclosure

Data collection from Powers Health, Munster, Indiana. Data analysis and manuscript creation done at the University of Illinois College of Medicine Rockford.

## Conflicts of Interest

Inna A. Husain is a consultant for Acclarent Inc. and the Vice President of Education for Reflux Raft. The other authors declare no conflicts of interest.

## Data Availability

The data that support the findings of this study are available from the corresponding author upon reasonable request.
